# *Neospora caninum* Activates p38 MAPK as an Evasion Mechanism against Innate Immunity

**DOI:** 10.3389/fmicb.2016.01456

**Published:** 2016-09-13

**Authors:** Caroline M. Mota, Ana C. M. Oliveira, Marcela Davoli-Ferreira, Murilo V. Silva, Fernanda M. Santiago, Santhosh M. Nadipuram, Ajay A. Vashisht, James A. Wohlschlegel, Peter J. Bradley, João S. Silva, José R. Mineo, Tiago W. P. Mineo

**Affiliations:** ^1^Laboratory of Immunoparasitology “Dr. Mário Endsfeldz Camargo,” Department of Immunology, Institute of Biomedical Sciences, Federal University of Uberlândia Uberlândia, Brazil; ^2^Department of Biochemistry and Immunology, School of Medicine of Ribeirão Preto, University of São Paulo Ribeirão Preto, Brazil; ^3^Department of Microbiology, Immunology and Molecular Genetics, University of California, Los Angeles, Los Angeles CA, USA; ^4^Department of Biological Chemistry and Institute of Genomics and Proteomics, University of California, Los Angeles, Los Angeles CA, USA; ^5^Molecular Biology Institute, University of California, Los Angeles, Los Angeles CA, USA

**Keywords:** *N. caninum*, immune response, p38/MAPk, evasion, IL-12

## Abstract

Due to the high prevalence and economic impact of neosporosis, the development of safe and effective vaccines and therapies against this parasite has been a priority in the field and is crucial to limit horizontal and vertical transmission in natural hosts. Limited data is available regarding factors that regulate the immune response against this parasite and such knowledge is essential in order to understand *Neospora caninum* induced pathogenesis. Mitogen-activated protein kinases (MAPKs) govern diverse cellular processes, including growth, differentiation, apoptosis, and immune-mediated responses. In that sense, our goal was to understand the role of MAPKs during the infection by *N. caninum*. We found that p38 phosphorylation was quickly triggered in macrophages stimulated by live tachyzoites and antigen extracts, while its chemical inhibition resulted in upregulation of IL-12p40 production and augmented B7/MHC expression. *In vivo* blockade of p38 resulted in an amplified production of cytokines, which preceded a reduction in latent parasite burden and enhanced survival against the infection. Additionally, the experiments indicate that the p38 activation is induced by a mechanism that depends on GPCR, PI3K and AKT signaling pathways, and that the phenomena here observed is distinct that those induced by *Toxoplasma gondii*’s GRA24 protein. Altogether, these results showed that *N. caninum* manipulates p38 phosphorylation in its favor, in order to downregulate the host’s innate immune responses. Additionally, those results infer that active interference in this signaling pathway may be useful for the development of a new therapeutic strategy against neosporosis.

## Introduction

*Neospora caninum* is an obligate intracellular protozoan from the phylum Apicomplexa, closely related to *Toxoplasma gondii*. This parasite has a worldwide distribution and causes relevant economic impact in dairy and beef industries (Cardoso et al., 2011) due to negative effects such as abortion or reproductive disorders in cattle, in addition to neuromuscular disease in dogs (Goodswen et al., 2012). Cattle are an important intermediate host species that may acquire the infection through horizontal transmission after ingestion of oocysts excreted by canine, which are the parasite’s definitive hosts. Infection may also occur during gestation, due to the immune response regulation, which leads to the recrudescence of chronic infections into subsequent parasitemia (Mineo et al., 2010a; Pinheiro et al., 2010). Afterward, the fetus may be infected by parasites that cross the placenta, causing abortions or congenital infections, depending on the period of gestation and parasite virulence (Eastick and Elsheikha, 2010).

The immune response against *N. caninum* is predominantly Th1-based, with the sequential production of interleukin-12 (IL-12), interferon gamma (IFN-γ) and nitric oxide (NO) by cells of the immune system, with a notable highlight to macrophages (MO), which underline the importance of cellular effector responses in the reduction of tissue parasitism and host survival, along with IgG2 immunoglobulin production (Abe et al., 2015; Donahoe et al., 2015; Hecker et al., 2015). IL-12 is a key cytokine that links the innate and adaptive compartments of the immune system, and is triggered by microbial products during early host–pathogen interactions (Aliberti, 2005; Yarovinsky et al., 2005; Debierre-Grockiego et al., 2007; Jenkins et al., 2010; Donahoe et al., 2015).

The usual production of this cytokine is initiated through recognition of highly conserved sets of molecular patterns (pathogen-associated molecular patterns, PAMPs) through a limited number of germline-encoded receptors in innate cells called pattern-recognition receptors (PRRs). The contact of these PAMPs with PRRs on macrophages triggers intracellular signaling pathways that result in the induction of an appropriated inflammatory response (Kawai and Akira, 2010). However, the host-specific intracellular signaling pathways triggered by innate recognition of *N. caninum* have not yet been fully elucidated. Previous studies demonstrated that initial *N. caninum* recognition includes TLR2, TLR3, and TLR11 (Jenkins et al., 2010; Mineo et al., 2010b; Beiting et al., 2014). Engagement of these receptors triggers activation of MyD88 or TRIF-dependent pathways, respectively, enhancing the immune response against this parasite (Mineo et al., 2009; Beiting et al., 2014). CCR5 is also a key player in the immune response against *N. caninum* through the production of cyclophilin, a parasite protein that modulates migration and activation of innate cells during the early phase of the infection (Mineo et al., 2010a; Abe et al., 2015).

The sustained synthesis of IL-12 in tissues and cells hosting Apicomplexan parasites is not controlled only by NF-κB, and also requires activation of MAPK pathways (Kim et al., 2005; Masek et al., 2006). MAPK pathways are composed by several kinases that activate an orchestrated cascade (MAP4K, the MAP3K, the MAP2K), which culminates in the phosphorylation of specific proteins of MAPK pathways that regulate the expression of sets of genes and effector proteins. The three most studied MAPK pathways are c-Jun-activated kinases (JNKs), extracellular signal-related kinases (ERKs) and p38 MAPK. MAPKs are activated by dual phosphorylation of the threonine and tyrosine residues, mediated by upstream MAPK kinases (MKK) (Kim et al., 2005; Yang et al., 2013). Usually, the ERK pathway induces growth factor signals, whereas the JNK and p38 pathways may be activated by a variety of extracellular stress signals (Cuadrado and Nebreda, 2010; Yang et al., 2013). Studies have been proposed that *T. gondii* induces phosphorylation of p38α MAPK in order to promote IL-12p70 production (Cuadrado and Nebreda, 2010). *T. gondii* secretes GRA24, a dense granule protein that has no ortholog expressed in *N. caninum*, that has the unique ability to trigger autophosphorylation and nuclear translocation of host p38, which correlates with the up-regulation of the transcription factors Egr-1 and c-Fos, and consequent synthesis of key proinflammatory cytokines, including IL-12 (Braun et al., 2013). Additionally, previous studies have reported that *T. gondii* also exploits heterotrimeric Gi-protein-mediated signaling to activate phosphoinositide 3-kinases (PI3Ks), leading to phosphorylation of PI3K and MAPK pathways, that results in inhibition of apoptosis and regulation of cytokine production (Kim and Denkers, 2006; Quan et al., 2013).

Within this context, the present study aimed to characterize the effect of MAPK signaling in the activation of immune responses against *N. caninum* and its antigens. Our results provide evidence that the parasite evades effective host responses through the activation of p38 MAPK-dependent G protein-coupled receptor (GPCR)/PI3K/AKT pathway.

## Materials and Methods

### Ethics Statement

All animal studies were approved by the animal research committee at UFU (Comitê de Ética na Utilização de Animais da Universidade Federal de Uberlândia – CEUA/UFU), under protocol number 029/12. All procedures including housing and welfare were carried out in accordance with the recommendations in the Guiding Principles for Biomedical Research Involving Animals of the International Council for Laboratory Animal Science (ICLAS), countersigned by the Conselho Nacional de Controle de Experimentação Animal (CONCEA; Brazilian National Consul for the Control of Animal Experimentation) in its E-book ^1^. The UFU animal facility (Centro de Bioterismo e Experimentação Animal – CBEA/UFU) is accredited by the CONCEA (CIAEP: 01.0105.2014) and Comissão Técnica Nacional de Biossegurança (CTNBio, Brazilian National Commission on Biossecurity; CQB: 163/02).

### Parasite and Antigens

Wild type (Nc-1) and genetically modified (NcLivΔHPT and NcLiv_GRA24-BirA^∗^) strains of *N. caninum* and *T. gondii* tachyzoites (PRU isolate) were maintained by serial passages in HeLa cell line (CCL-2, ATCC, Manassas, VA, USA), cultured in RPMI 1640 medium supplemented with 2 mM glutamine, 100 U/mL penicillin and 100 μg/mL streptomycin, at 37°C in 5% CO_2_ atmosphere. Parasite suspensions were obtained as previously described (Ribeiro et al., 2009). Briefly, tachyzoites were harvested by scraping off the cell monolayer after 2–3 days of infection, passed through a 26-gauge needle and centrifuged at low speed (45 × *g*) for 1 min at 4°C, for debris removal. The supernatant containing parasite suspension was collected, washed in phosphate buffered saline (PBS, pH 7.2), and the pellet was resuspended in PBS for antigen preparation.

*N. caninum* lysate antigen (NLA) was prepared as previously described (Ribeiro et al., 2009). Briefly, parasite suspension was treated with protease inhibitors (Roche Diagnostics GmbH, Mannheim, Germany) and then lysed by freeze-thaw cycles, followed by ultrasound cycles (60 H/30 s) on ice. After centrifugation (10,000 × *g*, 30 min, 4°C), the supernatant was collected, filtered on 0.22 μm membranes and its protein concentration determined by Bradford assay (Sigma–Aldrich, St. Louis, MO, USA). NLA aliquots were stored at -20°C until used *in vitro* assays.

Preparation of *N. caninum* excreted-secreted antigen (NcESA) was carried out as described elsewhere (Ribeiro et al., 2009). Freshly egressed tachyzoites (10^8^ parasites/ml) were washed twice in PBS, resuspended in Hanks saline solution and incubated for 30 min at 37°C, at mild agitation. Parasites were then centrifuged (720 × *g*, 10 min, 4°C), the supernatant was collected and again centrifuged (10,000 × *g*, 4°C, 30 min). The final supernatant was filtered on 0.22 μm membranes and its protein concentration determined by Bradford (1976). Different batches of NcESA were prepared and pooled together to obtain the required protein concentration. NcESA aliquots were stored at -20°C until use.

To exclude that experiments conduced with different strains and p38 inhibitors would generate distinct results, we have tested both parental *N. caninum* strains used (Nc-1 and NcLivΔHPT), well as both p38 specific chemical inhibitors (SB203580 – for *in vitro* use only; and SB239063 – preconized for *in vivo* use) and observed that there are no notable phenotype differences (Supplementary Figure 1).

### Differentiation of Bone Marrow-Derived Macrophages (BMDMs)

Bone-marrow stem cells were obtained from femurs and tibiae of 6- to 8-week-old wild-type and PI3K, MyD88 and CCR5 deficient C57BL/6 mice, according to previous description (Marim et al., 2010). Briefly, mice were euthanized and the bones were collected and flushed to extrude bone marrow. The cell suspension was used to generate bone marrow derived macrophages (BMDMs) using L929-cell conditioned medium (LCCM) as a source of granulocyte/macrophage colony stimulating factor. Cells were seeded in non-tissue culture treated petri dishes (Optilux, BD Biosciences) and incubated at 37°C in a 5% CO_2_ atmosphere. Four days after seeding the cells, an extra of fresh R20/30 (RPMI1640 supplemented with 20% FBS, 30% LCCM, 100 U/ml penicillin, 100 μg/mL streptomycin, and 2 mM L-glutamine) were added per plate. After 7 days, adherent cells were removed and analyzed by flow cytometry. Once the positive phenotype was confirmed (CD11b expression over 95%), BMDMs were plated at a concentration of 1.10^6^ cells/ml of RPMI, supplemented with 10% FBS, before some further experimental procedure.

### Measurement of MAPK Activation

Bone marrow derived macrophages were added to 12-well plate at a density of 1 × 10^6^ cells per ml and infected with live tachyzoites (Nc-1 or NcLivΔHPT; 1:1 ratio of parasites to cells). The cells were lysed in denaturation buffer 5x containing protease and phosphatase inhibitors (Roche), 0, 15, 30, 60, and 120 min post-infection.

Preparation of samples was performed according to the manufacturer’s protocol for adherent cells (BD Biosciences; Schubert et al., 2009). Briefly, cells were lysed and collected from the plates. Samples were then placed immediately in a boiling water bath for 5 min. Cell lysates were centrifuged at 15.000 × *g* for 5 min and supernatants were stored at -80°C until measurement. pJNK1/2 (T183/Y185), pp38 (T180/Y182), and pERK1/2 (T202/Y204) were quantitatively determined using antibodies from the multiplex Flex Set Cytometric Bead Array (pCBA, BD Biosciences). Serial dilutions (1/2 v/v) of the standards were prepared; cell lysates were (1/2 v/v) diluted using assay diluent and transferred to a FACS tube. Then, 25 μl of mixed capture beads were transferred to each tube. After a 3 h incubation at room temperature, 25 μL PE detection reagent was added and samples were incubated for another hour at room temperature. FACS tube was washed with buffer and after final centrifugation, 200 × *g* for 10 min, 150 μL wash buffer was added. Flow cytometric analysis was performed using a flow cytometer (FACSCantoII, BD Biosciences) and dedicated software (FCAP array v3.0, BD Biosciences). A total of 900 events were acquired following the protocol supplied. The minimum detection levels for each phospho-protein were: pJNK = 0.38 U/ml; pp38 = 0.64 U/ml; pERK = 0.64 U/ml.

### Western Blotting

For Western blotting, BMDMs were added to 12-well plate at a density of 1 × 10^6^ cells/ml and infected with *N. caninum* tachyzoites or stimulated with LPS or PMA. After 30 min, the cells were collected in lysis buffer containing a mixture of protease and phosphatase inhibitors (Roche). The lysates were resolved by SDS-PAGE and transferred onto nitrocellulose membranes. Blots were probed with the primary antibodies against p-p38, total p38, p-pERK1/2, total pERK1/2,p-JNK and total JNK (R&D Systems), followed by secondary antibodies conjugated to horseradish peroxidase (HRP). Target proteins were visualized by chemiluminescence for the detection of HRP activity (ChemiDoc XRS, Bio-Rad, Hercules, CA, USA). Additionally, whole-parasite lysates from parental and modified strains were separated by 12% SDS-PAGE. Samples were transferred to nitrocellulose overnight and probed with primary antibodies. For all secondary antibody incubations, HRP-conjugated goat anti-mouse or goat anti-rabbit antibodies were used at a 1:2000 dilution. Following secondary incubation, a chemiluminescent substrate was used for the detection of HRP activity.

### Phenotyping of Costimulatory Molecules (B7) and Major Histocompability Complex (MHC)

Bone marrow derived macrophages were added to 12-well plates at a density of 1 × 10^6^ cells/mL, pretreated for 3 h with p38 inhibitor (SB203580, 10 μM) or medium before antigenic stimulus (NLA, 10 μg/mL). After, 24 h of stimulated cells were labeled with antibodies conjugated to fluorochromes, following the protocol previously described (Mineo et al., 2010b). For these tests, we used the following commercial antibodies: CD11b-APC.Cy7 (clone M1/70), MHC II-APC (clone AF-120.1), MHC I-PE.Cy7 (clone AF-88.5), CD80-PE (clone 16-10A1), and CD86-FITC (clone GL1) (BD Biosciences). Phenotyping of BMDMs was performed in a flow cytometer (FACSCanto II, BD Biosciences) and the data was processed and analyzed using dedicated software (FlowJo, TreeStar, USA).

### Antigenic Recall Using Primed Spleen Cells

For measurement of *ex vivo* cytokine production, spleens retrieved from mice infected by *N. caninum* (5 × 10^6^ Nc-1 tachyzoites/mouse) or infected and treated with p38 inhibitor SB239063 (0.5 mg/Kg), along with its appropriated controls, were dissociated 30 days post infection (dpi) in RPMI medium and cell suspensions were washed in medium and added a lysis buffer (0.16 M NH4Cl and 0.17M Tris–HCl, pH 7.5), cells were washed again and resuspended in complete RPMI medium containing 10% FBS. Viable cells (2 × 10^5^ cells/200 μl/well) were cultured in triplicate in 96-well plates in the presence of antigen (NLA, 10 μg/ml), mitogen (Concanavalin A – ConA, 2.5 μg/ml), Nc-1 tachyzoites (1:10 parasite per cells) or medium alone and incubated at 37°C in 5% CO_2_. After 48 h, cell-free supernatants were collected and stored at -80°C for the measurement of cytokines.

### Challenge

*In vivo* experiments were conducted with two different parasite doses, depending on the aim of each experiment: (i) WT mice (at least six mice/group) were challenged intraperitoneally with lethal doses (1 × 10^7^ Nc-1 tachyzoites/mouse), treated or not for 5 days with the p38 inhibitor SB239063 (0.5 mg/Kg), in order to observe the effect of the p38 inhibitor treatment in the survival of the challenged mice. Animals were observed daily for morbidity and mortality during 30 dpi. (ii) In other sets of experiments, WT mice (six mice/group) were infected intraperitoneally with sub-lethal doses (5 × 10^6^ Nc-1 tachyzoites/mouse), treated or not for 5 days with SB239063 (0.5 mg/Kg), for the evaluation of immunological parameters and chronic phase parasite burden. After 7 days of infection, blood samples were collected and analyzed for the concentration of cytokines. After 30 days of infection, brain tissues and spleen cells were used for the quantification of chronic phase parasite burden and evaluation of cytokine production upon antigenic recall, respectively.

### Cytokine Quantification

Bone marrow derived macrophages were added to 96-well plate at a density of 1 × 10^6^ cells/mL. Stimulation was performed with live *N. caninum* tachyzoites (1:1 ratio of parasites to cells), NLA (10 μg/mL) or ESA (10 μg/mL). In some experiments, BMDMs were pretreated for 3 h with p38 inhibitor (SB203580, 10 μM), JNK inhibitor (SP600125, 10 μM), ERK1/2 inhibitor (PD98059, 10 μM), GPCRs inhibitor (Pertussis toxin, 200 ng/ml), PI3K inhibitor (Wortmannin, 500 nM), AKT inhibitor (AKT quinase VIII, 10 μM) or medium before infection. The concentration of each drug was chosen based on previously published manuscripts (Kim et al., 2005; Ruhland and Kima, 2009; Zong et al., 2012). p38 inhibition by SB203580 was verified in BMDMs by pCBA (Supplementary Figure 2). After, 24 h of infection or antigenic stimulus, the culture supernatant were collected for the measurement of IL-12p40 cytokine concentration. Quantification of IL-12p40 and IFN-γ in BMDMs and splenocytes was performed by ELISA, according to the manufacturer instructions (BD Biosciences). The assays were read at 450 nm and the OD values obtained were converted to pg/mL by the extrapolation of the standard curve (M2e plate reader, Molecular Devices, Sunnyvale, CA, USA). Serum of WT mice infected with Nc-1 strain (5 × 10^6^ tachyzoites/mouse), submitted or not to SB239063 treatment (0.5 mg/Kg), was collected after 7 dpi. The effect on the cytokine profile during SB239063 treatment was evaluated using a mouse Th1/Th2/Th17 cytometric bead array kit (CBA), according to the manufacturers’ instructions (BD Biosciences). The assays were read in a flow cytometer (FACSCanto II, BD Biosciences), and the concentration of each analyte was extrapolated from standard curves of recombinant cytokines. The minimum detection levels for each cytokine were: IL-2 = 0.1 pg/mL; IL-4 = 0.03 pg/mL; IL-6 = 1.4 pg/mL; IFN-γ = 0.5 pg/mL; TNF = 0.9 pg/mL; IL-17A = 0.8 pg/mL; IL-10 = 16.8 pg/mL.

### Determination of Parasite Burden

Brain parasite burden was determined by quantitative real-time PCR as previously described (Ribeiro et al., 2009), by the use of primer pairs (sense 3′-GCTGAACACCGTATGTCGTAAA-5′; antisense 3′-AGAGGAATGCCACATAGAAGC-5′) to detect the *N. caninum* Nc-5 sequence. DNA extraction was performed from 20 mg of murine brain tissues (Genomic DNA kit, Promega Co., USA) and parasite loads were calculated by interpolation from a standard curve of Nc-1 tachyzoite DNA included in each run (StepOne Plus, Applied Biosystems, USA). As a negative control, DNA obtained from brain tissues of non-immunized and unchallenged mice was analyzed in parallel.

### Generation of *GRA24* Knock-in Parasites

Targeting constructs were generated using the pNeoGra7-3xHA-BirA^∗^-HPT vector. Briefly, Type II GRA24 gene locus is defined by TGME49_230180 in the *T. gondii* genome database^2^ (version 6.0). The gene was cloned by PCR with engineered Nsi I 5′ sites and Not1 3′ sites. After amplification in a pJET vector, the *GRA24* insert was fused in frame with a plasmid using the same restriction sites (**Figure 5A**) (*manuscript in preparation*). The construct was linearized by Hind III restriction digest and 75 μg of the DNA was transfected by electroporation into NcLivΔHPT parasites. Stable transformants were selected using medium containing 25 μg/mL of mycophenolic acid plus 25 μg/mL of xanthine (for HXGPRT plasmids) and cloned by limited dilution in 96 well plates. To confirm proper *Gra24* gene targeting and expression, transgenic parasites were analyzed by IFA and the HA-tagged construct was shown to be co-localized in the parasitophorous vacuole with GRA2 (Supplementary Figure 3).

### Immunofluorescence Assay

For IFA, HFFs were grown to confluence on coverslips with medium containing or not 150 μM of biotin and infected with *N. caninum* parasites expressing HA-tag and BirA^∗^ fusions. After 36 h, the coverslips were fixed and processed for indirect immunofluorescence as previously described (Chen et al., 2015). The coverslips were mounted in Vectashield (Vector Labs) and viewed with an Axio Imager.Z1 fluorescence microscope (Zeiss).

### Affinity Capture of Biotinylated Proteins

HFF monolayers infected with parasites expressing BirA^∗^ fusions, or its respective parental line, were grown in medium containing 150 μM biotin for 24 h prior to parasite egress (Chen et al., 2015). Cells infected were collected, washed in PBS, and lysed in RIPA buffer (50 mM Tris [pH 7.5], 150 mM NaCl, 0.1% SDS, 0.5% sodium deoxycholate, 1% NP-40) supplemented with protease inhibitor cocktail (Roche) for 30 min on ice. Lysates were centrifuged for 15 min at 14,000 × *g* to pellet insoluble debris and then incubated with streptavidin magnetic beads (Pierce, USA), at room temperature for 4 h, under gentle agitation. Beads were collected using magnets and washed five times in RIPA buffer, followed by three washes in 8 M urea buffer (50 mM Tris-HCl [pH 7.4], 150 mM NaCl). Ten percent of each sample was boiled in Laemmli sample buffer, and eluted proteins were analyzed by Western blotting with streptavidin-HRP prior to mass spectrometry analysis.

### Mass Spectrometry

By sequential addition of Lys-C and trypsin proteases the purified biotinylated proteins bound to streptavidin beads were reduced, alkylated, and digested (Chen et al., 2015). The peptide mixture was desalted by the use of C18 tips and fractionated by a 75 μm inner diameter filter fused to silica capillary column with a 5 μm pulled electrospray tip, and packed in house with 15 cm of Luna C18 column with 3 μm reversed-phase particles. Delivering of the gradient was performed by an easy-nLC 1000 ultrahigh-pressure liquid chromatography (UHPLC) system (Thermo Scientific). Tandem mass spectrometry (MS/MS) spectra were collected on a Q-Exactive mass spectrometer (Thermo Scientific). Data analysis was done using the ProLuCID and DTASelect2 implemented in the Integrated Proteomics pipeline IP2 (Integrated Proteomics Applications, Inc., San Diego, CA, USA). Protein and peptide identifications were filtered using DTASelect and required a minimum of two unique peptides per protein and a peptide-level false-positive rate of less than 5%, as estimated by a decoy database strategy. Normalized spectral abundance factor (NSAF) values were calculated as previously described (Chen et al., 2015).

### Statistical Analysis

Differences amongst the groups were analyzed using ANOVA followed by Bonferroni multiple comparison post-tests to examine all possible pairwise comparisons, with few exceptions: (i) Comparisons involving parasite burden quantification by qPCR were analyzed by Kruskal–Wallis test followed by Dunn multiple comparison post-test; (ii) Student *t*-test was used for comparison between IgG1 and IgG2abc isotypes between different individual groups; (iii) The Kaplan–Meier method was applied to estimate the percentage of mice surviving at each time point after challenge and survival curves were compared using the Log-rank test. Analysis was carried out using GraphPad Prism 6.0 (GraphPad Software Inc., La Jolla, CA, USA). Values of *P* < 0.05 were considered statistically significant. Each experiment was independently conducted at least two times, and each condition was analyzed in triplicates, at least.

## Results

### *N. caninum* Induced Rapid Phosphorylation of p38 MAPK

**FIGURE 1:**
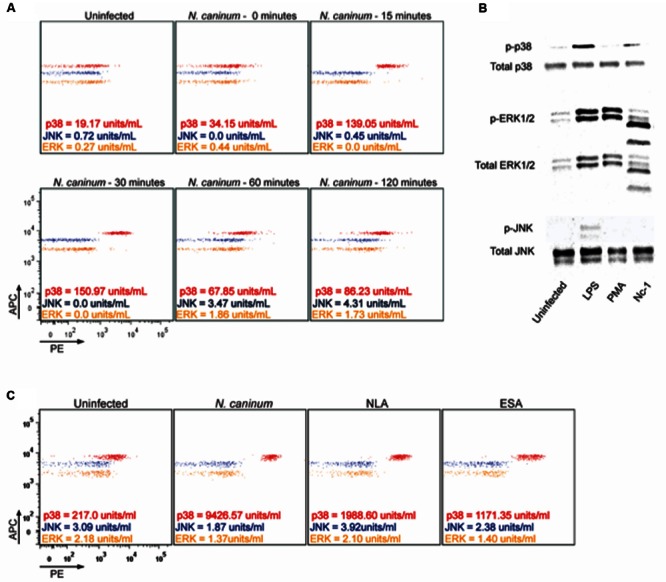
***Neospora caninum* induces phosphorylation of p38 MAPK in macrophages.** BMDMs (1 × 10^6^ cells/ml) were infected with live Nc-1 isolate tachyzoites of *N. caninum* (Nc, 1:1 parasite to cell ratio) or stimulated with NLA (10 μg/mL) or ESA (10 μg/mL) and incubated under controlled temperature and atmosphere. **(A)** Representative dot plots of the kinetics of MAPKs phosphorylation after exposure of BMDMs to Nc (0, 15, 30, 60, and 120 min post infection) by CBA; **(B)** Expression of the activated MAPKs after 30 min of infection by Western Blotting. Uninfected cells were used as negative controls and, as a positive control, BMDMs stimulated with 1 μg/mL of Lipopolysaccharides (LPS) or 100 nM of Phorbol-12-myristate-13-acetate (PMA); **(C)** Representative dot plots of the phosphorylation of the different MAPKs tested after stimuli with live *N. caninum* tachyzoites or its soluble lysate (NLA) and excreted/secreted (ESA) antigens by flow cytometry. Results are representative of three independent experiments, with at least three technical replicates in each, which were extrapolated in relation the standard curve and are expressed as units/mL.

To determine the effect of *N. caninum* infection on MAPK activation in macrophages, the phosphorylation of major MAPK signaling components was examined by CBA and Western Blotting (**Figure 1**). CBA experiments revealed that *N. caninum* induced strong phosphorylation of p38 MAPK (p-p38) between 15 and 30 min after contact with live parasites, with a tendency to reduce the reactivity after 60 min of infection. ERK1/2 and JNK did not show similar activation upon exposure to live parasites (**Figure 1A**). The same profile was observed by western blotting of BMDMs after 30 min of infection (**Figure 1B**).

We next evaluated MAPK activation in macrophages exposed to different antigenic stimuli. For that purpose, BMDMs were exposed to *N. caninum* tachyzoites, soluble lysate antigen (NLA) or excreted-secreted antigens (ESA) and analyzed after 30 min (**Figure 1C**). It was observed that all antigens induced p-p38, although the highest levels were observed after infection with live *N. caninum* tachyzoites.

In reference to those findings, we suggest that *N. caninum* activates predominantly p38 MAPK after infection of BMDMs, probably through a component of its excreted/secreted antigens.

### Parasite-Triggered p38 MAPK Activation Dampens Antigen Presentation Features of Macrophages

**FIGURE 2:**
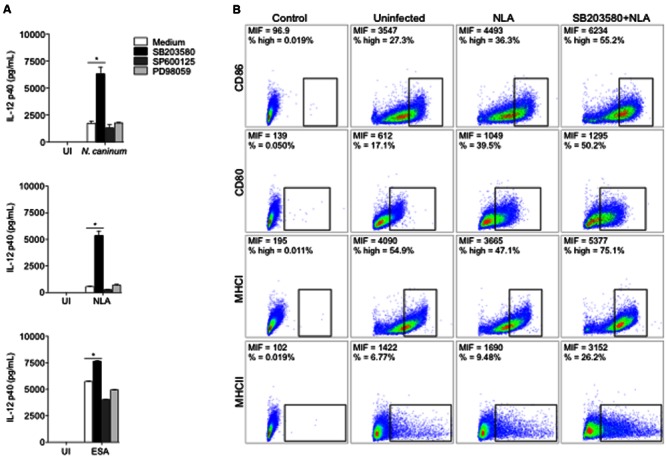
***Neospora*-induced p38 activation downregulates the production of IL-12 and the expression of antigen presentation molecules.** BMDMs (1 × 10^6^ cells/mL) were pretreated for 3 h with p38 inhibitor (SB203580, 10 μM), SAPK/JNK inhibitor (SP600125, 10 μM), ERK1/2 inhibitor (PD98059, 10 μM). **(A)** Cells were infected with *N. caninum* Nc-1 strain (Nc; 1:1 parasite to cell ratio) or stimulated with NLA (10 μg/mL) or ESA (10 μg/mL) or medium alone (UI). After 24 h, supernatants were collected and analyzed for IL-12p40 concentration. Results were expressed as mean ± SEM. ^∗^Indicates statistically significant differences between untreated cells and SB203580-treated cells, upon antigenic stimulation; *P* < 0.05, assessed by ANOVA followed by Bonferroni multiple comparison post-test to examine all possible pairwise comparisons. Results are representative of at least five independent experiments, with five technical replicates each. **(B)** BMDMs were stimulated with NLA (10 μg/mL) for 24 h and then checked for population positivity (%) and mean intensity of fluorescence (MIF) of surface markers CD80, CD86, MHCI, and MHCII. Results are representative of two independent experiments, with three technical replicates each, and dot plots were represented as percentage of positives cells, where warmer colors indicate higher cell densities.

We first verified the role of p38, JNK and ERK in IL-12p40 production in *N. caninum*-infected BMDMs. Inhibition of p38 MAPK by SB203580 induced IL-12p40 upregulation after 24 h of infection with live parasites or its components (**Figure 2A**), while chemical inhibition of ERK 1/2 and JNK did not yield notable differences in relation to controls.

To characterize the phenotype of *N. caninum-*infected BMDMs, the expression of B7 co-stimulatory (CD80 and CD86) and Major Histocompability Complex (MHCI and MHCII) molecules were observed, upon MAPK chemical inhibition. Macrophages pretreated with SB203580 and exposed to NLA showed higher expression of B7 and MHC molecules, percentage wise or by mean intensity of fluorescence (MIF), if compared to uninfected or infected untreated cells (**Figure 2B**). Similar results were observed in experiments with live tachyzoites (Supplementary Figure 4).

Based on these experiments, we have observed that p38 MAPK activation in infected macrophages is associated with pronounced decrease in IL-12p40 production, alongside with the expression of MHC and B7 co-stimulatory molecules.

### p38 Inhibition Is Effective in Therapeutic and Immune-Prophylactic Strategies against *N. caninum* through the Upregulation of Cellular Responses

**FIGURE 3:**
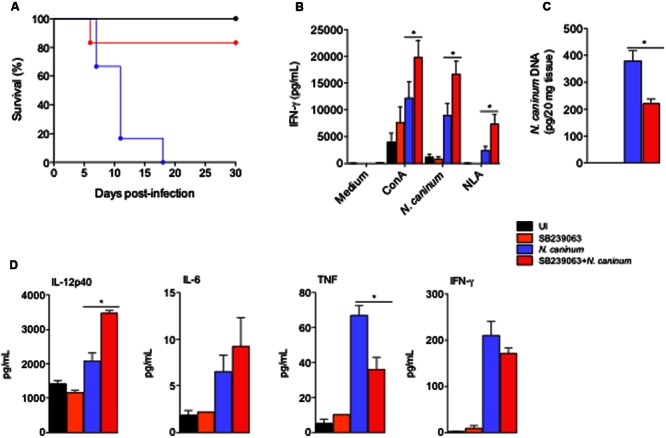
**Treatment with p38 inhibitor improved immune responses and increased survival of mice infected with *N. caninum*.** WT mice were treated or not with p38 inhibitor (SB239063, 0.5 mg/Kg) for 5 days post infection in order to observe survival. As controls, mice were inoculated with SB239063 alone (inhibitor control), or PBS (uninfected - UI). **(A)** Survival curves (six mice/group). **(B)** IFN-γ production during antigenic recall of spleen cells recovered from mice after 30 days of sub-lethal infection. Spleen cells (1 × 10^6^/ml; five technical replicates) were cultured in the presence of mitogen (ConA, 2.5 μg/ml); live Nc-1 tachyzoites (1:10 parasite to cell ratio), soluble lysate antigen (NLA, 10 μg/ml) or medium alone. Supernatants were collected after 48 h and analyzed by ELISA. ^∗^Indicates statistically significant differences (*P* < 0.05) between untreated mice and SB239063-treated mice, upon antigenic stimulation, assessed by ANOVA followed by Bonferroni multiple comparison post-test to examine all possible pairwise comparisons. Results are representative of two independent experiments, with five technical replicates each. **(C)** Brain parasite load after 30 days of sub-lethal infection with *N. caninum* in the different test groups (five mice/group), analyzed by real-time PCR. ^∗^Indicates statistically significant differences (*P* < 0.05) between untreated mice and SB239063-treated mice, assessed by Kruskal–Wallis followed by Dunn’s multiple comparison post-test. Results are representative of two independent experiments, with three technical replicates each. **(D)** Cytokine production in the serum of mice after 7 days of sub-lethal infection treated or not with SB239063, analyzed by ELISA and CBA. Results are expressed as mean ± SEM of cytokine levels (five mice/group), extrapolated in relation to each assays’ standard curve, and representative of two independent experiments, with two technical replicates/each. ^∗^Indicates statistically significant differences (*P* < 0.05) between untreated mice and SB239063-treated mice; assessed by ANOVA followed by Bonferroni multiple comparison post-test to examine all possible pairwise comparisons.

To assess whether p38 inhibition had a therapeutic effect *in vivo*, we first infected groups of mice with a lethal dose (1 × 10^7^ Nc-1 tachyzoites/mouse) for 100% of the animals (LD100), in order to evaluate clinical parameters as survival (**Figure 3A**). Infected mice treated with p38 inhibitor SB239063 did not show significant differences in the body weight or temperature loss, if compared to the other groups (*P* > 0.05, data not shown). Strikingly, the treatment rescued 84% of the mice from LD100, yielding a significant statistical increase in the survival rate (*P* = 0.0042), if compared to the untreated group of animals.

We also examined whether the treatment with p38 inhibitor would affect the antigenic recall in chronically infected mice. With that intent, we observed the production of IFN-γ in *ex vivo* stimulation of spleen cells obtained from the different groups of mice during chronic phase of the infection (30 days after inoculation) with sub-lethal tachyzoite doses (5 × 10^6^ Nc-1 tachyzoites/mouse). Spleen cells from SB239063-treated and infected mice produced statistically higher concentration of IFN-γ upon stimulation with the mitogen Concanavalin A (ConA, *P* = 0.0029), live tachyzoites (1/10 parasites/cell ratio, *P* < 0.0001) and NLA (20 μg/mL, *P* = 0.0014), if compared to cells extracted from untreated and infected littermates (**Figure 3B**; Means and SEM are represented in Supplementary Table 1). In agreement, the group of mice treated with the p38 MAPK inhibitor presented a significantly reduced concentration of parasite genomic DNA in the central nervous system (*P* < 0.0001) than the infected mice group (**Figure 3C**). In another set of experiments, mice were infected with sub-lethal doses of tachyzoites and treated for 5 days with p38 inhibitor SB239063, in order to test how inhibition of p38 MAPK *in vivo* would affect the outcome of disease, through immune and parasitological parameters. We observed that mice infected and treated with the chemical inhibitor had higher serum concentration of pro-inflammatory cytokines as IL-12p40 than the untreated group (**Figure 3D**) during the acute phase of infection (7 days after inoculation).

To assess whether p38 inhibition would also be a target for immune-prophylactic strategy, we immunized mice with NLA (50 μg/mouse) and used SB239063 as adjuvant in the preparation of the test group. We observed that mice immunized with NLA plus SB239063 presented mean reduction of 75% in the concentration of *N. caninum* genomic DNA in the central nervous system compared to the PBS (*P* < 0.05, Supplementary Figure 5). We also sought to observe if p38 MAPK activation interfered with the production of specific antibodies against *N. caninum*. With that intent, we measured the production kinetics of specific IgG antibodies and its subclasses IgG1 and IgG2abc, in the serum of mice 0, 15, 30, and 45 days after immunization. The ELISA assays determined that mice immunized with SB239063 presented similar production of specific antibody against *N. caninum* (Supplementary Figure 6), indicating that p38 signaling blockade does not affect antibody production after immunization against *N. caninum*.

### GPCR, PI3K, and AKT Are Required for *Neospora*-Induced p38 MAPK Downregulation of IL-12 Production

**FIGURE 4:**
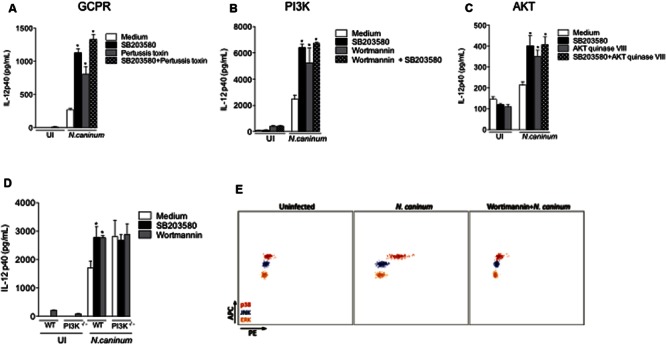
***N. caninum* infection activates p38 MAPK signaling through GPCR and PI3K/AKT pathways.** Bone marrow-derived macrophages (BMDMs) of WT mice were pretreated with inhibitors to **(A)** GPCR (Pertussis toxin, 200 ng/ml), **(B)** PI3K (Wortmannin, 500 nM), **(C)** AKT (AKT inhibitor VIII, 10 μM), in addition or not to p38 inhibitor (SB203580, 10 μM), for 3 h, and sequentially infected by *N. caninum* live tachyzoites (Nc-1; 1:1 ratio of parasites to cells), or left uninfected (UI). After 24 h of infection, the supernatants were collected for IL-12p40 measurement. **(D)** BMDMs generated from WT mice and genetically deficient littermates in PI3K (PI3K^-/-^) were inhibited by SB203580 and/or Wortmannin, prior to infection with *N. caninum* live tachyzoites (Nc-1; 1:1 parasite to cell ratio) for the measurement of IL-12p40. ^∗^Indicates statistically significant differences between untreated cells and SB203580-treated cells, upon antigenic stimulation; *P* < 0.05, assessed by ANOVA followed by Bonferroni multiple comparison post-test to examine all possible pairwise comparisons. Results are representative of at least five independent experiments, with five technical replicates each. **(E)** Representative dot plots of p38 phosphorylation in WT BMDMs inhibited or not by Wortmannin for 3 h, and subsequently infected with *N. caninum* (Nc-1; 1:1 ratio) for 30 min, measured by CBA. Results are representative of three independent experiments, with three technical replicates/each.

To define the molecular mechanisms that underlie the regulatory effects of parasite mediated p38 activation, we tested distinct signaling pathways with known (direct or indirect) ability of inducing p38 phosphorylation. *Neospora*-infected BMDMs were treated with chemical/biological inhibitors of G-coupled protein receptors (GPCRs, pertussis toxin, **Figure 4A**), PI3K (Wortmannin, **Figure 4B**) and AKT (AKT inhibitor VIII, **Figure 4C**), combined or not with p38 inhibition (SB203580), in order to observe a possible additive effect in the IL-12p40 production. That experimental approach led to the observation that those inhibitors yielded exactly the same increase in cytokine production as SB203580, as well as the double inhibition of infected BMDMs, strongly suggesting that *Neospora*-induced p38 activation was dependent on upstream signaling of GPCR, PI3K, and AKT. To confirm that PI3K played a crucial role in the activation of p38 MAPK, we observed the cytokine production of BMDMs genetically depleted of PI3K (PI3K^-/-^) and infected by *N. caninum* (**Figure 4D**). In this set of experiments, we observed that the absence of PI3K induced similar increase of IL-12p40 production as that induced by inhibition of p38 MAPK. Also, the pretreatment of the chemical inhibitors SB203580 and Wortmannin in PI3K^-/-^ BMDMs did not alter the increased IL-12p40 production after exposure to tachyzoites. In order to provide direct evidence to corroborate that *Neospora*-induced p38 activation was dependent of PI3K signaling, we also observed that PI3K inhibition by Wortmannin abrogated p38 MAPK phosphorylation in BMDMs after 30 min of infection with *N. caninum* tachyzoites (**Figure 4E**).

We also assessed whether CCR5, MyD88, NF-κB, AP-1, mTOR, and JAK2 were involved in p38 activation in BMDMs during *N. caninum* infection, through the measurement of the concentration of IL-12p40 after 24 h. As previously shown by our group (Mineo et al., 2009), genetic deficiency in MyD88 (MyD88^-/-^) completely abolished IL-12 production after exposure to live parasites (Supplementary Figure 7A). Genetically deficient BMDMs in CCR5 (CCR5^-/-^) or treatment with specific inhibitors to JAK2 (Tyrphostin AG490), NF-κB (CAPE), AP-1 (Tanshinone) or mTOR (Rapamycin) did not yield significant differences in cytokine production after infection, if compared to WT or untreated controls. Therefore, we conclude that the phenomena herein described, induced by p38 activation, are independent of those molecules (Supplementary Figures 7B–F).

### *N. caninum*-Triggered p38 Activation Is Induced by a Distinct Mechanism than *T. gondii*’s GRA24 Protein

**FIGURE 5:**
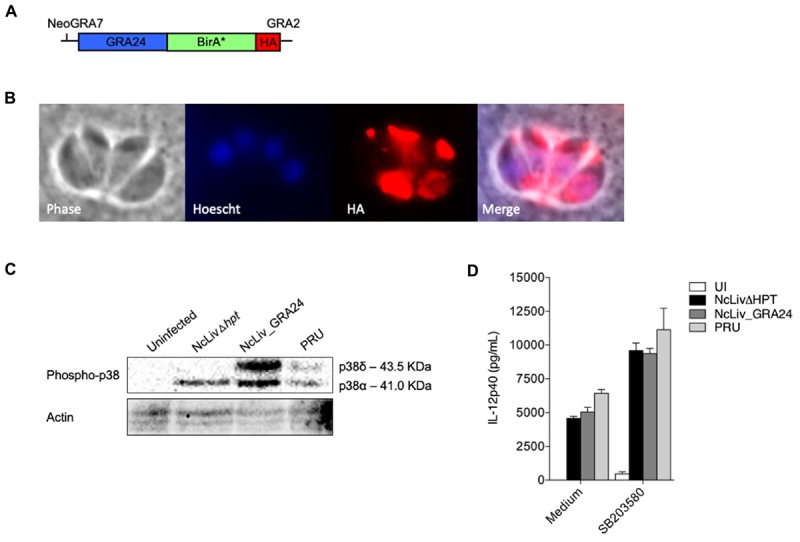
***N. caninum*-induced p38 phosphorylation in macrophages is triggered by a distinct mechanism than *T. gondii*’s GRA24 protein. (A)** Diagram of the expression cassette encoding GRA24 fused to BirA^∗^, plus a 1C-terminal 3 HA epitope tag, driven by the *N. caninum* GRA7 promoter; **(B)** IFA of GRA24-BirA^∗^-expressing parasites, grown for 48 h. GRA24-BirA^∗^ localizes to the parasitophorous vacuole. Red, mouse anti-HA antibody; Blue, Hoescht; **(C)** BMDMs (1 × 10^6^ cells/ml) were infected with live tachyzoites of *N. caninum* and *T. gondii* (NcLivΔHPT, NcLiv_GRA24, PRU; 1:1 parasite to cell ratio). After 30 min or 18 h, the cells were lysed and submitted to Western blot of p38 phosphorylation. Results are representative of at least two independent experiments. **(D)** BMDMs (1 × 10^6^ cells/ml) were pretreated for 3 h with p38 inhibitor (SB203580, 10 μM) and infected with live tachyzoites of *N. caninum* and *T. gondii* (NcLivΔHPT, NcLiv_GRA24, PRU; 1:1 ratio) for 24 h. The supernatants were collected and the concentration of IL-12p40 was measured by ELISA. Results were expressed as mean ± SEM, and are representative of at least two independent experiments, with five technical replicates each.

To assess whether the mechanisms herein described are similar to those driven by *T. gondii*’s type II exclusive protein GRA24 (TgGRA24), we have genetically modified *N. caninum* tachyzoites to express this secreted antigen. For that purpose, we have generated a construct containing TgGRA24 with a C-terminal hemagglutinin (HA) tag plus BirA^∗^, driven by *N. caninum GRA7* promoter (**Figure 5A**). TgGRA24 was integrated into the genome of NcLivΔHPT (parental) parasites, and localization of the gene fusion was assessed by immunofluorescence assay (IFA) using BirA/streptavidin interaction, as previously described (Chen et al., 2015). As expected, IFA showed that tagged GRA24 was correctly targeted to the dense granules, as it colocalized with GRA2 protein dispersion in the parasitophorous vacuole (data not shown), and the BirA^∗^-streptavidin complex further corroborated this pattern (**Figure 5B**), demonstrating that the construct for heterologous protein expression was successful.

Although MS experiments with *N. caninum* GRA24-BirA^∗^ expressing tachyzoites did not retrieve biotinylated p38 MAPK within its results, we continued to investigate whether the mechanism behind p38 pathway triggered by *N. caninum* shares common features with those described for TgGRA24, BMDMs were infected for 30 min and 18 h by parental (NcLivΔHPT), TgGRA24+ *N. caninum* (NcLiv_GRA24) or type II *T. gondii* (PRU) tachyzoites. As seen in **Figure 5C**, NcLivΔHPT induced a significantly less robust p38 activation compared to parasites that expressed type II TgGRA24 (NcLiv_GRA24 and PRU), independently if observed after 30 min or 18 h of exposure to the tachyzoites. Finally, we assessed if the addition of TgGRA24 in *N. caninum* tachyzoites would further enhance IL-12 production. For that purpose, cells were treated with p38 inhibitor SB203580 and infected with NcLivΔHPT, NcLiv_GRA24 or PRU tachyzoites. This assay demonstrated that all tested parasites induced similar cytokine production, as inhibition of p38 MAPK induced higher IL-12p40 production in all infected BMDMs, if compared to infected and untreated cells (**Figure 5D**). These results show that TgGRA24 does not further negatively interfere on IL-12p40 production in macrophages infected with *N. caninum*, demonstrating that the mechanisms herein reported—downregulation of IL-12 by activation of the p38 MAPK pathway by Neospora’s antigens—are distinct from those previously described for *T. gondii* (Braun et al., 2013), although it also makes us speculate whether the ability to evade innate immune responses through the GCPR/PI3K/AKT/p38 pathway is preserved between the parasites.

## Discussion

Control of neosporosis involves the introduction of standard biosecurity measures; actually control programs are usually based on decreasing the vertical transmission in cattle by reducing the number of seropositive herd and/or decreasing the risk of horizontal transmission of *N. caninum*, mainly by the control of domestic definitive host population as a source of oocyst contamination. Afterward, different control measures have been suggested, however, the strategies have been ineffective (Dubey et al., 2007; Reichel et al., 2015). Thus, the increase in knowledge about the host immune response after infection by this parasite is essential to develop effective vaccine and treatment protocol.

Macrophages are the most important phagocytic cells in mammals and play a key role in the pathogens detection and elimination. Together with DCs, they provide the first line of cell-mediated defense and are pivotal in controlling the initial dissemination and/or growth of intracellular parasites (Donahoe et al., 2015; Hecker et al., 2015). Macrophage functions include synthesis of cytokines, such as IL-12, co-stimulatory molecules and presentation of antigens to T cells through the MHC that trigger adaptive immune responses, as well as antimicrobial mechanisms such as phagocytosis and NO synthase (Mineo et al., 2009; Monney and Hemphill, 2014). Earlier studies have convincingly shown that intracellular parasites like *T. gondii* and *Leishmania* sp. induces the MAPK pathway as modulatory mechanism in the IL-12 production in macrophages, and that this response is dependent of the phosphorylation of p38 MAPK (Rincón et al., 1998; Gigley et al., 2009; Yang et al., 2010; Dion et al., 2011; Quan et al., 2015). Therefore, MAPK pathway plays a key role in the control of intracellular infection, resulting in regulation of inflammatory cytokines that compose the Th1 immune responses. Therefore, we assessed for the first time the role of MAPK pathway in *N. caninum*-infected BMDMs, and we observed that *N. caninum* induced early p38 MAPK phosphorylation, as previously described in infection of macrophages by other Apicomplexa parasites (Kim et al., 2005; Horton et al., 2011; Klotz et al., 2011). We also observed that the robust p38 phosphorylation was opposed to little to absent induction of ERK1/2 and JNK. This conclusion maybe drawn regardless of the technique applied, since western blotting and CBA yielded similar results.

We analyzed if distinct antigenic fractions of *N. caninum* would be capable of inducing of the same p38 activation profile than live tachyzoites, and found that all antigens tested (NLA and ESA) were able to induce the activation of p38. Apicomplexan parasites are able to actively invade host cells using the action of apical secretory organelles named the micronemes, rhoptries, and dense granules. The proteins impacts host signaling pathways to serve as “master regulators” of the immune system (Melo et al., 2011). Thus, the induction of p38 here observed could be induced by ESA, since recent work by others has emphasized that early phase parasite secretion of kinases and phosphatases co-opt host cells by interfering with their signaling pathways (Dubremetz, 2007; Hajagos et al., 2012; Jensen et al., 2013; Bougdour et al., 2014). Braun et al. (2013) identified GRA24 as a strong agonist of host p38-alpha. However, there are no apparent GRA24 orthologs within the *N. caninum* genome. The use of *N. caninum* as a heterologous system for the expression of foreign genes such as *T. gondii* has been explored, and it may prove to be a tool for the parasite factors identification that are involved in the phenotypic differences stated between these apicomplexa parasites (Lei et al., 2014). The results showed that *N. caninum* expressing TgGRA24 induced higher activation of the subunit p38-alpha than the parental line, although it is noteworthy to point out that the BirA^∗^ bared within the construct was not tested to whether it would interfere with the observed phenomena. We actually speculate that *N. caninum* may activate a distinct subunit of p38 MAPK, since the other biological phenotypes assessed were distinct than that induced by NcLiv_GRA24 or PRU. Previous studies reported about some surface genes and secreted proteins, which are known to control virulence and host interactions in *T. gondii* infection, are altered in *Neospora* genome with different expression and functionality, which imply that there have been significant changes in the evolution of host-interacting genes between these species (Reid et al., 2012; Ramaprasad et al., 2015; Silmon de Monerri and Weiss, 2015). Hence, the pursuit for such antigenic targets with potential p38 activation is desired and should be better investigated.

The p38 pathway is associated with cytokine production, inflammation, cell growth, differentiation, and death (Terrazas et al., 2011). Studies with other infectious parasites, as *Leishmania*, have considered the role of MAP kinases in the regulation of IL-12 production (Yang et al., 2007, 2010; Ruhland and Kima, 2009), however, there is no clear consensus on the role of p38 activation in the regulation of this key cytokine. In the present study, we have identified that p38 plays a crucial role in dampening the cellular immune responses of mice infected by *N. caninum*, since mice treated with specific p38 inhibitors had increased pro-inflammatory cytokine production, lower chronic phase parasite burden and higher survival than untreated mice. Also, the use of p38 inhibitors as an adjuvant in vaccine protocols could block the immunosuppressant effects of *N. caninum* antigens, by significantly reducing the parasite load of challenged mice. It is noteworthy that inhibition of p38 alone during the immunization schemes was able to completely abrogate chronic phase parasitism, fact which should be further explored in future experiments. An additional remark should be made toward the possible direct effects of the p38 inhibitor in the tachyzoites, during *in vivo* treatment. Wei et al. (2002) reported that SB203580 or SB202190 treatment in *T. gondii*-infected fibroblasts significantly inhibited intracellular tachyzoite replication. Also, in a *Leishmania major* model, mice vaccinated with heat-killed *L. major* plus CpG and SB203580 elicited complete protection against the infection (Horton et al., 2011), and a similar protection was observed when p38 MAPK blockade inhibited the replication of *Plasmodium falciparum* in human erythrocytes cultured *ex vivo* (Brumlik et al., 2011). Thus, this work corroborates and adds new information to the literature that p38 MAPK inhibitors may be a potential active principle to improve treatment and vaccines against intracellular protozoa.

The precise mechanism underlying the phenomena described in this work is yet to be unveiled. Our data show that one of the interactions between *N. caninum* and macrophages is induced through activation of the GPCR/PI3K/AKT pathway, which by its turn induces p38 phosphorylation and consequent blockade of crucial mediators required for an accurate immune response against the parasite. In agreement with this finding, studies demonstrated that inhibition of PI3K/Akt signaling resulted in increased IL-12 production against infections by intracellular parasites, although these studies were inconclusive on how this signaling pathway could be mediated by p38 MAPK (Ruhland and Kima, 2009; Quan et al., 2015). In order to describe this pathway as that responsible for this downregulation of the innate immune responses against the parasite, we have also checked if other known signaling pathways were involved. None yielded results that agree with the phenotype observed. To rationalize our choices for the pathways, we initially assessed MyD88, a major adaptor protein for Toll-like receptor (TLR) signaling, which our group previously described as critical for acute resistance to *N. caninum* infection (Mineo et al., 2009), especially regarding the induction of IL-12p40. We have also assessed the participation of CCR5, a GCPR-dependent chemokine receptor that is known to induce MyD88-independent inflammatory cell migration during *N. caninum* infection (Mineo et al., 2010a; Bonfá et al., 2014; Abe et al., 2015). During *T. gondii and N. caninum* infections, parasite-derived cyclophilin 18 induces IL-12 and IFN-γ production by binding to CCR5. Moreover, *N. caninum* cyclophilin caused CCR5-dependent migration of murine and bovine cells (Aliberti et al., 2000; Ibrahim et al., 2009; Kameyama et al., 2012). We also showed that none of the classical transcription factors is directly involved with upstream phosphorylation of p38 MAPK, since IL-12p40 production was not affected by the inhibition of JAK2, NF-κB, AP-1 and mTOR. In this sense, p38 MAPK may regulate alternative transcription factors, as the ATF family - nuclear factor-erythroid 2-related factor 2 (Nrf2), Egr-1 and c-Fos which induce a wide variety of physical, chemical and biological effect that should be further investigated (Chakrabarti et al., 2014; Chi et al., 2015).

## Conclusion

*N. caninum* downregulates, possibly through parasite secreted-excreted antigens, the signaling pathway composed of PI3K/AKT and p38 MAPK pathways downstream of GPCR signaling. Our results indicate that *N. caninum* manipulates those pathways in its favor, in order to evade the host’s innate immune responses, increasing acute phase replication and consequently chronic phase parasite burden. In that sense, that pathway may be a potential target for therapeutic intervention against neosporosis.

## Author Contributions

Conceived and designed the experiments: CM, TM, and PB. Performed the experiments: CM, AO, MD-F, MS, SN, and AV. Analyzed the data: CM, TM, and PB. Contributed reagents/materials/analysis tools: CM, TM, FS, JW, PB, JdS, and JM. Wrote the paper: CM, TM, and PB.

## Conflict of Interest Statement

The authors declare that the research was conducted in the absence of any commercial or financial relationships that could be construed as a potential conflict of interest.

## Footnotes

1 http://www.mct.gov.br/upd_blob/0238/238271.pdf

2 www.toxodb.org
