# Corrigendum: *Neospora Caninum* Activates p38 MAPK as an Evasion Mechanism against Innate Immunity

**DOI:** 10.3389/fmicb.2019.00548

**Published:** 2019-03-26

**Authors:** Caroline M. Mota, Ana C. M. Oliveira, Marcela Davoli-Ferreira, Murilo V. Silva, Fernanda M. Santiago, Santhosh M. Nadipuram, Ajay A. Vashisht, James A. Wohlschlegel, Peter J. Bradley, João S. Silva, José R. Mineo, Tiago W. P. Mineo

**Affiliations:** ^1^Laboratory of Immunoparasitology “Dr. Mário Endsfeldz Camargo,” Department of Immunology, Institute of Biomedical Sciences, Federal University of Uberlândia, Uberlândia, Brazil; ^2^Department of Biochemistry and Immunology, School of Medicine of Ribeirão Preto, University of São Paulo, Ribeirão Preto, Brazil; ^3^Department of Microbiology, Immunology and Molecular Genetics, University of California, Los Angeles, Los Angeles, CA, United States; ^4^Department of Biological Chemistry and Institute of Genomics and Proteomics, University of California, Los Angeles, Los Angeles, CA, United States; ^5^Molecular Biology Institute, University of California, Los Angeles, Los Angeles, CA, United States

**Keywords:** *N. caninum*, immune response, p38/MAPk, evasion, IL-12

In the original article, there was an error. We have recently discovered the contamination of a batch of *Neospora caninum*, Liverpool isolate (NcLiv), used by our labs over the last few years as a base strain for genetic modification assays, with a *Toxoplasma gondii* knockout strain. This issue has been made public by our research groups during the retraction of another article (https://doi.org/10.1038/s41598-018-28052-2). Regarding the article herein referred, this issue has compromised a single set of experiments, which are displayed in Figure 5.

**Figure 5:**
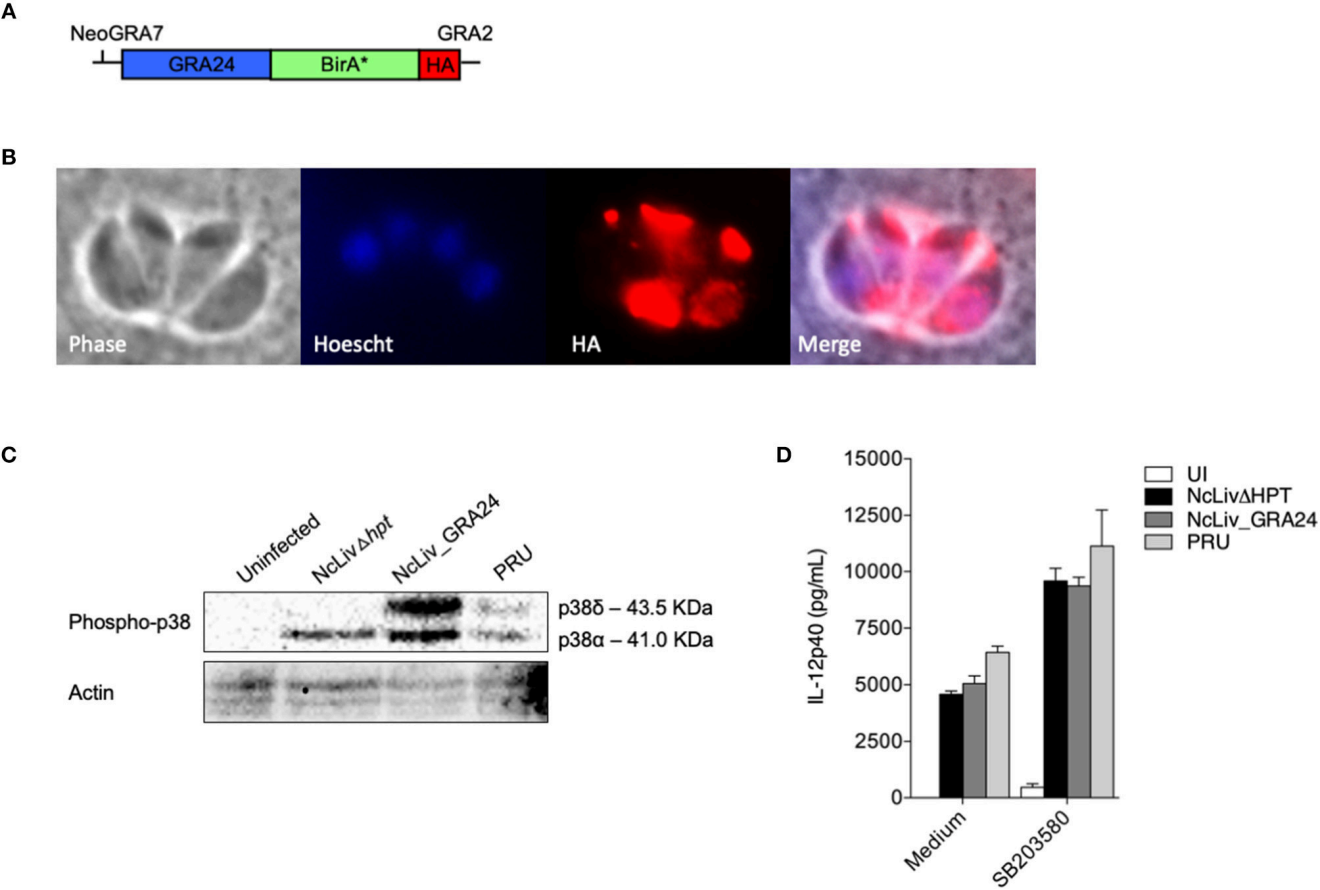
*N. caninum*-induced p38 phosphorylation in macrophages is triggered by a distinct mechanism than *T. gondii*'s GRA24 protein. **(A)** Diagram of the expression cassette encoding GRA24 fused to BirA^*^, plus a 1C-terminal 3 HA epitope tag, driven by the *N. caninum* GRA7 promoter; **(B)** IFA of GRA24-BirA^*^-expressing parasites, grown for 48 h. GRA24-BirA^*^ localizes to the parasitophorous vacuole. Red, mouse anti-HA antibody; Blue, Hoescht; **(C)** BMDMs (1 × 10^6^ cells/ml) were infected with live tachyzoites of *N. caninum* and *T. gondii* (NcLivΔHPT, NcLiv_GRA24, PRU; 1:1 parasite to cell ratio). After 30 min or 18 h, the cells were lysed and submitted to Western blot of p38 phosphorylation. Results are representative of at least two independent experiments. **(D)** BMDMs (1 × 10^6^ cells/ml) were pretreated for 3 h with p38 inhibitor (SB203580, 10 μM) and infected with live tachyzoites of *N. caninum* and *T. gondii* (NcLivΔHPT, NcLiv_GRA24, PRU; 1:1 ratio) for 24 h. The supernatants were collected and the concentration of IL-12p40 was measured by ELISA. Results were expressed as mean ± SEM, and are representative of at least two independent experiments, with five technical replicates each.

All the other experiments of the originally published article were not affected by this error, since other parasite stocks/isolates were used, and the data is reproducible and sound.

In order to address this issue, we have repeated, with success, the experiments contained in Figure 5 using the proper background: HPT depletion of NcLiv, construction of parasites expressing *T. gondii*'s GRA24 (type II) using the NcGRA7 promoter, proper localization of the protein in the vacuoles of infected cells, compatible MAPK p38 phosphorylation and induction of differential IL-12 production; with the exception of the identification of biotinylated p38 MAPK by MS.

Although unfortunate, this experiment was a mere additional control of the ortholog gene expressed in *N. caninum*, since GRA24-p38 MAPK interactions had been extensively described elsewhere (DOI: 10.1084/jem.20130103).

We truly believe that scientific integrity is the basis of the advancement of knowledge.

A correction has therefore been made to the **Results**, section ***N. caninum*-Triggered p38 Activation Is Induced by a Distinct Mechanism than** ***T. gondii*'s GRA24 Protein**. Paragraph two has been removed and the following paragraph has been corrected to:

“Although MS experiments with *N. caninum* GRA24-BirA^*^ expressing tachyzoites did not retrieve biotinylated p38 MAPK within its results, we continued to investigate whether the mechanism behind p38 pathway triggered by *N. caninum* shares common features with those described for TgGRA24, BMDMs were infected for 30 min and 18 h by parental (NcLivΔHPT), TgGRA24+ *N. caninum* (NcLiv_GRA24) or type II *T. gondii* (PRU) tachyzoites. As seen in Figure 5C, NcLivΔHPT induced a significantly less robust p38 activation compared to parasites that expressed type II TgGRA24 (NcLiv_GRA24 and PRU), independently if observed after 30 min or 18 h of exposure to the tachyzoites. Finally, we assessed if the addition of TgGRA24 in *N. caninum* tachyzoites would further enhance IL-12 production. For that purpose, cells were treated with p38 inhibitor SB203580 and infected with NcLivΔHPT, NcLiv_GRA24 or PRU tachyzoites. This assay demonstrated that all tested parasites induced similar cytokine production, as inhibition of p38 MAPK induced higher IL-12p40 production in all infected BMDMs, if compared to infected and untreated cells (Figure 5D). These results show that TgGRA24 does not further negatively interfere on IL-12p40 production in macrophages infected with *N. caninum*, demonstrating that the mechanisms herein reported—downregulation of IL-12 by activation of the p38 MAPK pathway by Neospora's antigens—are distinct from those previously described for *T. gondii* (Braun et al., 2013), although it also makes us speculate whether the ability to evade innate immune responses through the GCPR/PI3K/AKT/p38 pathway is preserved between the parasites.”

The corrected Figure 5 and legend appears below:

The authors apologize for this error and state that this does not change the scientific conclusions of the article in any way. The original article has been updated.
