# Supplementary material for: Neospora caninum Activates p38 MAPK as an Evasion Mechanism against Innate Immunity
Source: Front Microbiol. 2016 Sep 13;7:1456. doi: 10.3389/fmicb.2016.01456 (PMC5020094; doi:10.3389/fmicb.2016.01456)
Supplement: Supplementary file 1 [file Data_Sheet_1.PDF]

Supplementary Material

Neospora caninum activates p38 MAPK as an evasion mechanism against innate immunity

Caroline Martins Mota[a], Ana Carolina Moraes Oliveira[a], Marcela Davoli-Ferreira[b], Murilo Vieira Silva[a], Fernanda Maria Santiago[a], Santhosh M. Nadipuram[c], Ajay A. Vashisht[d], James A. Wohlschlegel[d], Peter John Bradley[c, e], João Santana da Silva[b], José R. Mineo[a], Tiago W. P. Mineo[a,*]

*Corresponding author:
E-mail: tiago.mineo@ufu.br
Tel: +55 34 3225-8666
Postal Address: Laboratory of Immunoparasitology "Dr. Mário Endsfeldz Camargo", ICBIM/UFU, Av. Amazonas s/n, 4C01, 38405-320, Campus Umuarama, Uberlândia, MG, Brazil

Supplementary Table 1. Mean IFN-γ production (pg/mL) during antigenic recall of spleen cells recovered from mice after 30 days of sub-lethal infection.

In vivo treatment		***In vitro stimuli***							
		**Medium**		**ConA**		***N. caninum***		**NLA**	
		Mean	*SEM*	*Mean*	*SEM*	*Mean*	*SEM*	*Mean*	*SEM*
	**Medium**	2,46	0,81	1991,12	79,21	1171,31	51,55	28,41	28,41
	**SB239063**	2,44	1,58	7667,45	169,9	804,55	31,7	0,00	0,00
	N. caninum	147,1	52,93	11318,04	228,49	12010,82	184,91	2203,55	60,19
	**SB239063+*N. caninum***	66,13	27,68	15375,13	164,85	15007,33	184,72	6414,94	52,51

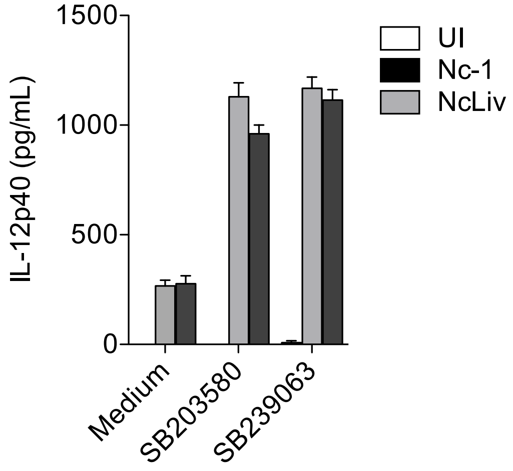

Supplementary Figure 1. Distinct *Neospora* strains yield similar IL-12p40 production upon p38 inhibition. BMDMs (1x10^6 cells/mL) were pretreated for 3h with p38 inhibitor (SB203580 or SB239063, 10 μM) and infected with live tachyzoites of Nc-1 or NcLivΔHPT isolates of *N. caninum* (1:1 ratio of parasites to cells) or left in medium alone (UI). After 24h, supernatants were collected for IL-12p40 measurements. Results were expressed as mean ± SEM (five biological replicates, two technical replicates). No statistically significant differences were observed among cells infected with either parasites (Nc-1 or NcLivΔHPT) or pretreated by either specific inhibitors (SB203580 or SB239063).

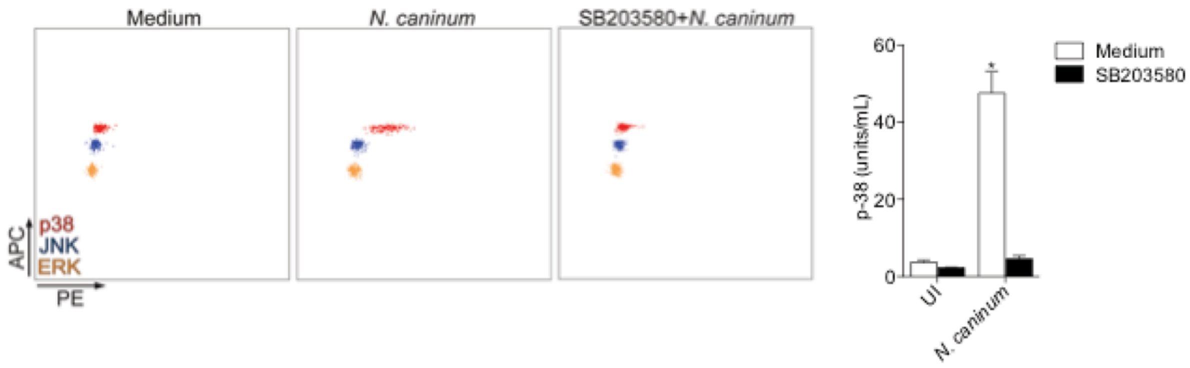

Supplementary Figure 2. p38 inhibition by SB203580 was efficient in BMDMs infected by *Neospora*. BMDMs (1x10^6 cells/mL) were pretreated or did not for 3h with p38 inhibitor (SB203580, 10 μM) and infected for 30 minutes with live tachyzoites of Nc-1 isolates of *N. caninum* (1:1 ratio of parasites to cells) or left in medium alone (UI). The p38/MAPK phosphorylation after exposure of BMDMs to Nc-1 by CBA was showed by representative dot plots (A) and bars graphic (B). Results are representative of three independent experiments, and were extrapolated in relation the standard curve and are expressed as units/mL. The values are expressed as mean ± SEM (Five biological replicates, two technical replicates; *Indicates statistically significant differences between untreated and SB203580-treated cells; $P < 0.05$).

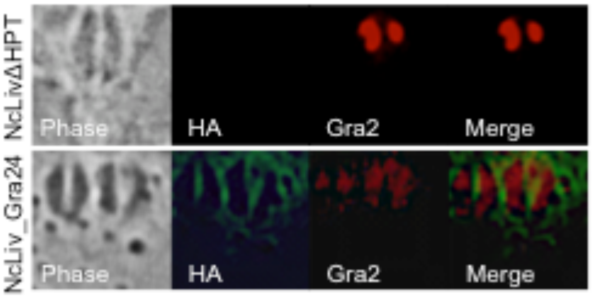

Supplementary Figure 3. Colocalization of type II *T. gondii* GRA24 construct to the parasitophorous vacuole in fibroblasts infected with transgenic *Neospora*. IFA of GRA24-expressing parasites grown for 24 h in medium. GRA24-HA (green) constructs were localized around the parasitophorous vacuole, while GRA2 (red) is closely associated to the dividing tachyzoites. Green, rabbit anti-HA antibody; red, mouse anti-IgG Alexa 594; blue, DAPI.

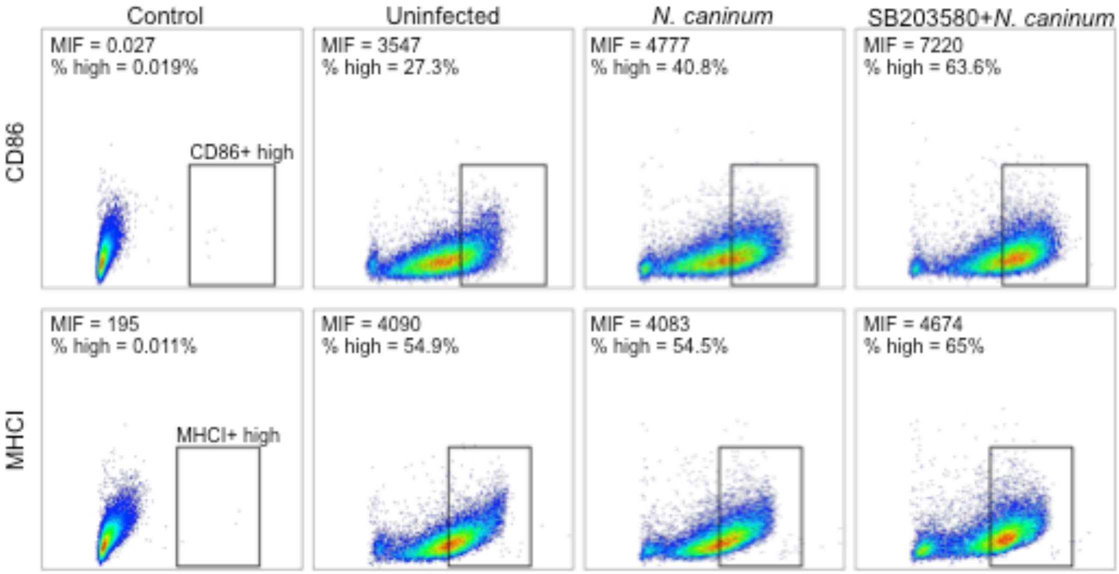

Supplementary Figure 4. Live *N. caninum* tachyzoites downregulate the expression of antigen presentation molecules. BMDMs ($1x10^6$ cells/mL) were pre-treated for 3h with p38 inhibitor (SB203580, 10 μM), and later infected with *N. caninum* Nc-1 strain (1:1 parasite to cell ratio). BMDMs were checked after 24h for population positivity (%) and mean intensity of fluorescence (MIF) of surface markers CD86 and MHCI. Results are representative of two independent experiments (with three biological replicates/each) and dot plots were represented as percentage of positives cells, where warmer colors indicate higher cell densities.

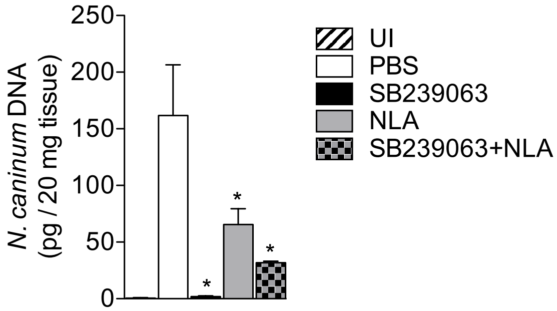

Supplementary Figure 5. Mice immunized with p38 inhibitor used as adjuvant presented lower chronic phase parasite burden. Groups of WT mice (6 animals/group) were immunized with *Neospora* lysate antigen (NLA) associated or not with p38 inhibitor (SB239063+NLA) as adjuvant. As controls, mice were immunized with NLA alone (antigen control), SB239063 alone (inhibitor controls) or PBS (infection control). After 45 days, immunized mice were challenged with 5 x 10^6 tachyzoites (Nc-1 strain) for the analysis of brain parasite load at 30 days post infection. Results were expressed as mean ± SEM. *Indicate statistically significant differences ($P < 0.05$) between the infected sham-immunized group (PBS) and immunized groups (SB239063, NLA or SB239063+NLA), assessed by Kruskal-Wallis followed by Dunn's multiple comparison post-test. Results are representative of two independent experiments, with three technical replicates each.

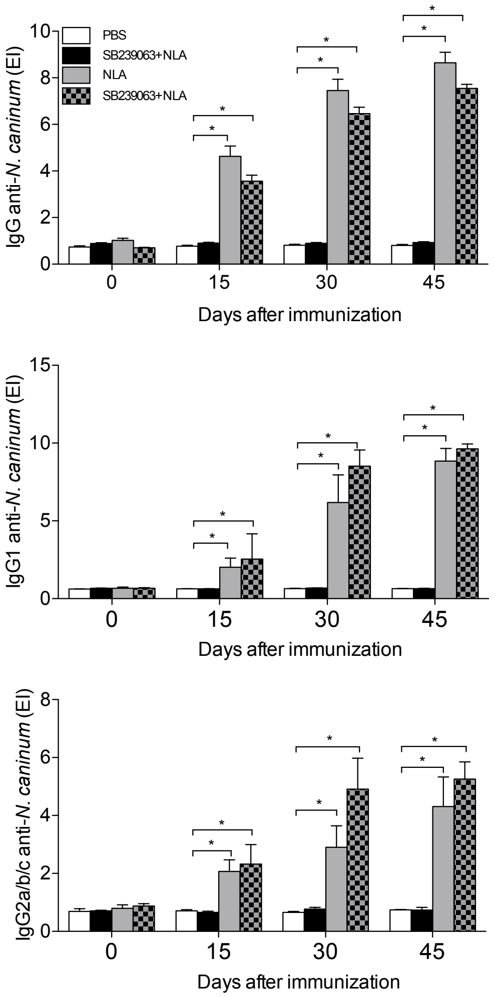

Supplementary Figure 6. Immunization with p38 inhibitor does alter specific antibody production. Groups of WT mice (6 animals/group) were immunized with *Neospora* lysate antigen (NLA) associated or not with p38 inhibitor (SB239063+NLA) as adjuvant. As controls, mice were inoculated with NLA alone (antigen control), SB203580 alone (inhibitor control) or PBS (infection control). Serum samples were collected at 0, 15, 30, and 45 days after immunization. Levels of total IgG, IgG1 and IgG2abc anti-*N. caninum* were determined by ELISA and results are expressed as mean ± SEM (*P < 0.05, Student t test).

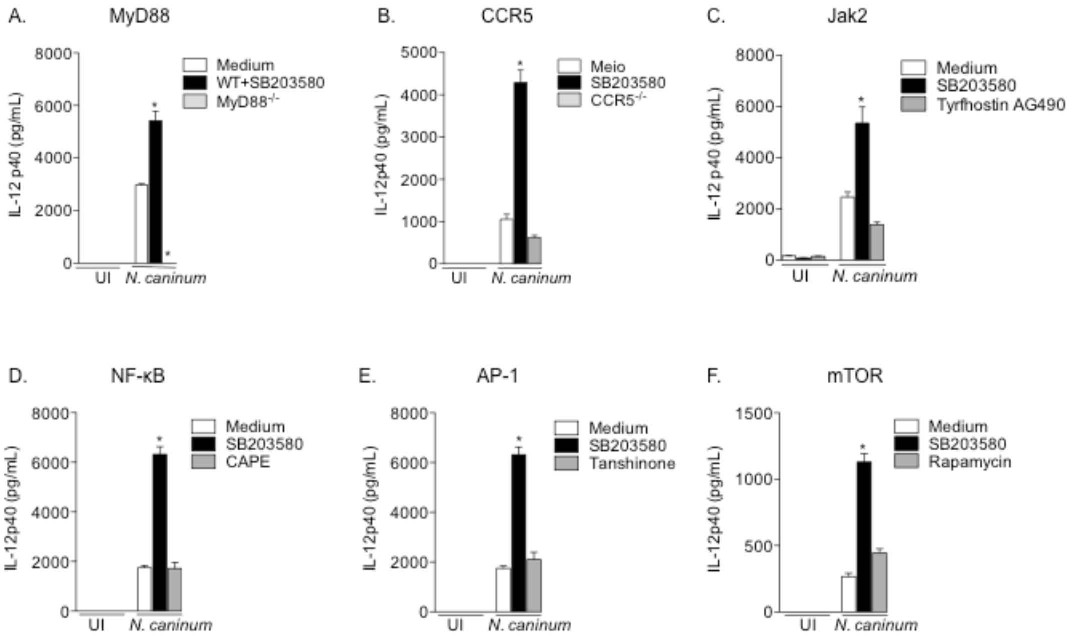

Supplementary Figure 7. *N. caninum*-induced p38 activation is independent of MyD88, CCR5, JAK2, NF-κB, AP-1 and mTOR. Bone marrow-derived macrophages (BMDMs) from wild type C57BL/6 and genetically deficient littermates were pretreated with inhibitors during 3 hours and later infected with *N. caninum* tachyzoites (Nc-1; 1:1 ratio of parasites to cells). After 24 hours of infection the supernatants were collected for the measurement of IL-12p40. (A) WT and MyD88$^{-/-}$ BMDMs, pretreated with p38 inhibitor (SB203580, 10 μM); (B) WT and CCR5$^{-/-}$ BMDMs, pretreated with SB203580; (C) WT BMDMs pretreated with JAK2 inhibitor (Tyrphostin AG490, 10 μM) and SB203580; (D) WT BMDMs pretreated with NF-κB inhibitor (CAPE, 10 μM) and SB203580; (E) WT BMDMs pretreated with AP-1 inhibitor (Tanshinone, 10 μg/ml) and SB203580; (F) WT BMDMs pretreated with mTOR inhibitor (Rapamicyn, 1 μM) and SB203580. Results are representative of three independent experiments and are expressed as mean ± SEM. *Indicate statistically significant differences ($P < 0.05$) among the infected (*N. caninum*) and untreated cells (medium) versus infected, WT or genetically deficient, treated cells, upon antigenic stimulation; $P < 0.05$, assessed by ANOVA followed by Bonferroni multiple comparison post-test to examine all possible pairwise comparisons. Results are representative of at least five independent experiments, with five technical replicates each.
